# Preparation and Characterization of Zinc Ferrite and Gadolinium Iron Garnet Composite for Biomagnetic Applications

**DOI:** 10.3390/ma17122949

**Published:** 2024-06-17

**Authors:** Bárbara Costa, João Carvalho, Sílvia Gavinho, Tânia Vieira, Jorge Carvalho Silva, Paula I. P. Soares, Manuel A. Valente, Sílvia Soreto, Manuel Graça

**Affiliations:** 1 i3N and Department of Physics, University of Aveiro, Campus Universitário de Santiago, 3810-193 Aveiro, Portugal; barbaracostaa@ua.pt (B.C.); silviagavinho@ua.pt (S.G.); mav@ua.pt (M.A.V.); mpfg@ua.pt (M.G.); 2 i3N/CENIMAT, Physics Department, NOVA School of Science and Technology, Campus de Caparica, NOVA University Lisbon, 2829-516 Caparica, Portugal; ts.vieira@fct.unl.pt (T.V.); jcs@fct.unl.pt (J.C.S.); 3 i3N/CENIMAT, Science Materials Department, Faculty of Sciences and Technology, Nova University of Lisbon, 2829-516 Caparica, Portugal; pi.soares@fct.unl.pt

**Keywords:** cancer, magnetic hyperthermia, gadolinium iron garnet, SAR, zinc ferrite

## Abstract

Cancer is a major worldwide public health problem. Although there have already been astonishing advances in cancer diagnosis and treatment, the scientific community continues to make huge efforts to develop new methods to treat cancer. The main objective of this work is to prepare, using a green sol–gel method with coconut water powder (CWP), a new nanocomposite with a mixture of Gd_3_Fe_5_O_12_ and ZnFe_2_O_4_, which has never been synthesized previously. Therefore, we carried out a structural (DTA-TG and X-ray diffraction), morphological (SEM), and magnetic (VSM and hyperthermia) characterization of the prepared samples. The prepared nanocomposite denoted a saturation magnetization of 11.56 emu/g at room temperature with a ferromagnetic behavior and with a specific absorption rate (SAR) value of 0.5 ± 0.2 (W/g). Regarding cytotoxicity, for concentrations < 10 mg/mL, it does not appear to be toxic. Although the obtained results were interesting, the high particle size was identified as a problem for the use of this nanocomposite.

## 1. Introduction

According to the World Health Organization, there were approximately 20 million new cases and 10 million deaths in 2020 worldwide due to cancer [1]. In 2040, it is estimated that there will be nearly 30 million diagnoses and 16 million deaths from cancer worldwide [2]. The evolution of science has led to a better understanding of the mechanisms behind cancer pathophysiology. Despite the visible decline in mortality, the increasing incidence of cancer justifies the continuous development of new treatment methods for the different types of cancer [3].

Since Richard Feynman’s lecture “There’s Plenty of Room at the Bottom” (1959), the advances of nanotechnology towards biomedical applications have undergone a major revolution in the diagnosis and treatment of cancer [4]. The combination of therapeutic and diagnostic (theranostics) in the same nanoparticle is an emerging tool that intends to act as a precise and personalized approach to cancer treatment [5]. However, the lack of knowledge on certain issues, such as the biological response to nanoparticles and their elimination in an organism, limits their use in clinical applications [5].

Magnetic nanoparticles (MNPs) are defined as nanostructures that possess at least one dimension on the nanoscale, with remarkable magnetic properties [4]. Their high surface-to-volume ratio [6] is responsible for their outstanding magnetic properties. In accordance with B. Issa et al. [7,8], the primary characteristics that they must have to be used for biomedical applications are biocompatibility and non-toxicity; a particle size distribution between 10 and 200 nm [9]; a high saturation magnetization (M_s_) to provide easy control of the particles in the blood through a moderate external magnetic field; and the possibility of better targeting of the pathologic tissue. By controlling the size, material, and coating of the MNPs, it is possible to improve and modify their properties considering the biomedical application in question.

In magnetic hyperthermia (MH) therapy, the magnetic heating efficiency of MNPs is a parameter that is extremely relevant. To evaluate their performance, specific parameters are usually used, such as specific heat absorption rate (SAR). SAR is defined as the capacity that a certain magnetic material has to generate heat. It is used to characterize the efficiency of heating a magnetic material through its absorption of energy during the exposition to an alternating magnetic field (AMF). In recent years, most of the studies have been focused on improving the SAR value of the MNPs for MH therapy use. Different methods have been used, such as controlling the particle core size, shape, composition, and surface shell, and selecting specific magnetic materials [10]. According to Liu et al. [11], due to the correlation between the decreasing of M_s_ as the size decreases, and knowing that SAR is proportional to M_s_, it is possible to conclude that SAR value increases with the increasing size of MNPs. Thus, MNPs with a higher M_s_ are desirable for effective magnetic loss [11].

Gadolinium nanoparticles (GdNPs) were first presented as a Magnetic Resonance Imaging (MRI) contrast agent by Carr et al. in 1984 [12]. Since then, MRI as well as other methods of medical imaging commonly employ this metal lanthanide as a contrast agent [12,13]. Due to its seven unpaired electrons and delayed electronic relaxation, the trivalent cation is regarded as a hard acid and is therefore employed in MRI [12]. In addition to its use in MRI, this element has drawn interest for various biomedical uses, most notably magnetic hyperthermia [14,15]. Jiang P. et al. [16] synthesized gadolinium-doped iron oxide nanoparticles with an elevated SAR value.

Spinel ferrite nanoparticles, such as MFe_2_O_4_ (where M = divalent metal ions, such as Co^2+^, Ni^2+^, Zn^2+^, Mn^2+^), are commonly used as MNPs for MH due to their astonishing chemical and physical properties [16,17]. Very good chemical stability, enhanced saturation magnetization, and high electrical resistivity are some of the outstanding properties that characterize them [17]. Zinc ferrite (ZnFe_2_O_4_) is a material that shows a lot of potential to be used in the biomedical field [18]. Bulk ZnFe_2_O_4_ has a normal spinel structure in which Zn^2+^ cations occupy the tetrahedral positions, while the octahedral positions are occupied by Fe^3+^ cations. However, when the dimensions are reduced to the nanoscale, it is described that the Zn^2+^ cations are distributed both on tetrahedral and octahedral sites, leading to their partially inverse spinel structure [19,20,21]. Among its different applications, zinc ferrite is seen as “the potential alternative material” [21], regarding its properties. In terms of biomedical applications, due to the lack of knowledge on the biological response of the organism and toxicity of zinc ferrite nanoparticles, there are only a few available clinical studies where only zinc ferrite was explored for MH. Somvanshi S. et al. [18] synthesized zinc ferrite nanoparticles using chemical coprecipitation and further functionalization with oleic acid. The obtained results showed superparamagnetic behavior, with a M_s_ of 25.5 emu/g. According to these outcomes, it is possible to conclude that zinc ferrite has the potential to be employed as a material for cancer treatment through MH.

Being aware of the advantages of green synthesis methods, new methods have been described by the scientific community as promising, cheaper, and sustainable ways to produce nanoparticles. Thus, based on its basic features and rich composition, the coconut water powder (CWP)-assisted sol–gel method has been described as a successful method to produce different MNPs. Some of the examples described in the literature are the synthesis of LiFe_5_O_8_ [22], Y_2_O_3_:Eu^3+^ [23], Y_2_O_3_:Nd^3+^ [24], niobium oxides [25], SrFe_12_O_19_ [26], and BaFe_12_O_19_ [27]. The main advantages of using this proteic sol–gel method based on coconut water are (1) low-cost method; (2) higher concentration of sugars, promoting the gelation process; (3) material widely and easily available around the world, being available at an industrial scale; (4) the promotion of a homogeneous distribution of the precursor ions due to the presence of proteins and amino acids that have the ability to bind with metal ions; and (5) importance as a renewable source [25].

The main purpose of this work is to develop and characterize structurally, morphologically, and magnetically a new nanocomposite of zinc ferrite (ZnFe_2_O_4_) and gadolinium iron garnet (Gd_3_Fe_5_O_4_). The aim is to synthesize this nanocomposite through a green synthesis sol–gel method using coconut water powder. This nanocomposite is intended to be used for MH treatment. Therefore, this nanocomposite’s SAR and biocompatibility are evaluated. 

## 2. Materials and Methods

### 2.1. Nanocomposite Synthesis

The coconut water powder (CWP)-assisted sol–gel method was used to prepare an efficient and novel nanocomposite of Gd_3_Fe_5_O_12_ and ZnFe_2_O_4_ able to be used for magnetic hyperthermia, as described by Teixeira S. et al. [22]. For Gd_3_Fe_5_O_4_, we used iron (III) nitrate (Fe(NO_3_)_3_·9H_2_O) (Merck KGaA, Darmstadt, Germany, ≥99.0%) and gadolinium nitrate hydrate (Gd(NO_3_)_3_·H_2_O) (Merck KGaA, Darmstadt, Germany, ≥99.0%), and for ZnFe_2_O_4_, we used iron (III) nitrate (Fe(NO_3_)_3_·9H_2_O) and zinc nitrate hexahydrate (Zn(NO_3_)_2_·6H_2_O) (Merck KGaA, Darmstadt, Germany, ≥99.9%) as precursors, respectively. 

**Figure 1:**
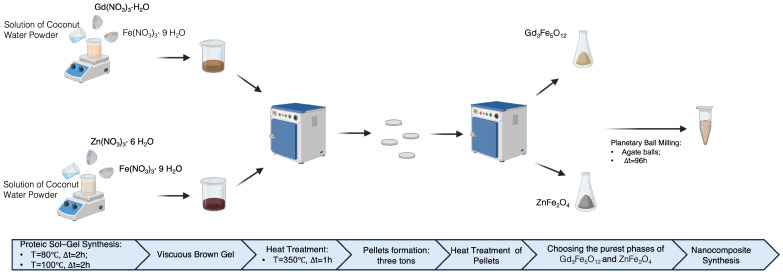
Schematic representation of the nanocomposite synthesis process.

First, each compound was synthesized individually. Then, after ensuring the best phase of each ferrite, Gd_3_Fe_5_O_12_ and ZnFe_2_O_4_ powders were mixed with a planetary ball mill to obtain the nanocomposite. For both Gd_3_Fe_5_O_12_ and ZnFe_2_O_4_, we applied the following steps (Figure 1):

(1) The metal precursors were dissolved into a CWP solution with a concentration of 0.58 mol/dm^3^, the critical micelle concentration (whose determination is explained in Section 3), and mixed with a magnetic stirrer in two steps:
  (a) At T = 80 °C, for 2 h;
  (b) At T = 100 °C, for 2 h.
(2) The obtained viscous brown gel was heat-treated at 350 °C for 1 h to remove the solvent;
(3) Preparation of pellets of Gd_3_Fe_5_O_12_ and ZnFe_2_O_4_ powders 10 mm in diameter and with a thickness of 2 mm, approximately, with three tons applied;
(4) The pellets were then submitted to different heat treatments using different values of dwell time and heating rate. The cooling process was performed according to the furnace’s thermal inertia when the power was switched off;
(5) After a structural analysis with X-ray diffraction, the purest phase of both Gd_3_Fe_5_O_12_ and ZnFe_2_O_4_ was chosen. To perform this analysis, the pellets were ground with the help of a mortar and pestle;
(6) The best phase of each compound was mixed with a planetary ball mill, the Pulverisette 7. The planetary ball mill was used due to its high efficiency in the process of mixing different materials [28].

Finally, the composite was obtained and characterized structurally, morphologically, magnetically, and biologically. 

### 2.2. Structural and Morphological Characterization

A structural and morphological analysis was performed to characterize structurally and morphologically the samples. The differential thermal analysis (DTA) and thermogravimetric analysis (TG) were conducted with Hitachi STA7300 equipment (Tokyo, Japan) in a nitrogen atmosphere with a flux of 200 mL. The analysis was performed with a heating rate of 5 °C/min in a range of temperatures from room temperature up to 1400 °C.

To characterize the crystalline structure of the samples, X-ray diffraction (XRD) was used. Therefore, Panalytical AERIS equipment from Malvern Panalytical (Westborough, MA, USA) with CuKα radiation with a 2θ angle (10–60°), a wavelength of 1.54060 Å, and at 40 kV and 15 mA was used. The XRD was performed using the powders obtained from the crushed pellets for each sample. The crystalline phases were identified with X’Pert HighScore Panalytical software version 5.2, which contains the database of the Joint Committee for Powder Diffraction Standards–International Center for Diffraction Data (JCPDS).

Scanning electronic microscopy (SEM) is a widely used technique that enables us to analyze the microstructure morphology. Thus, the TESCAN Vega3 SB (Warrendale, PA, USA) was used with an accelerating beam voltage of 30 kV. Carbon deposition was performed to ensure the samples’ conductivity. Using ImageJ 1.52v, the average grain size was determined.

### 2.3. Magnetic Characterization

A vibrating sample magnetometer (VSM), the Cryofree model from Cryogenic (London, UK), was used to measure the magnetization (M) of the samples vs. the magnetic field (H) up to 50 kOe. The values of M and H were measured in a range of temperature from −258.15 to 26.85 °C.

The magnetic hyperthermia assays were performed using a D5 series from the nB nanoscale Biomagnetics (Zaragoza, Spain). The samples were exposed to an alternating current magnetic field of 24 kAm^−1^, with a frequency of 388 Hz for 10 min. Each sample was immersed in 1 mL of ultra-pure water and was ultra-sonicated before each measurement.

In MH therapy, the specific heat absorption rate parameter (SAR) is used to evaluate the magnetic heating efficiency of MNPs. SAR is calculated using the following equation:

(1) $$SAR=\frac{m_{l}\times c_{l}+m_{Fe}\times c_{NP}}{m_{l}+m_{Fe}}\times \left(\frac{dT}{dt}\right)$$

where $\frac{dT}{dt}$ is given by the variation of temperature within a certain period of time, $c_{l}$ is the specific heat of the liquid, $c_{NP}$ is the specific heat of the magnetic material, $m_{l}$ is the fluid mass, and $m_{Fe}$ is the mass concentration of the magnetic element in the solution [8].

### 2.4. Cytotoxicity Analysis

The cytotoxicity assays were performed according to the ISO 10993-5:2009 standard “Biological evaluation of medical devices—Part 5: Tests for in vitro cytotoxicity” [29]. Due to the presence of gadolinium in human bones and its importance in bone regeneration [30], the SaOs-2 cell line (human osteosarcoma, ATCC HTB-85) was chosen as a cellular model of cells from bone.

The extracts were produced by placing 20 mg of each of the samples in 1 mL of complete culture medium (McCoy’s 5 A from Sigma-Aldrich (St. Louis, MO, USA), catalog number M4892; supplemented with 2.2 g/L sodium bicarbonate, Sigma-Aldrich, catalog number S5761; 1% penicillin/streptomycin from Gibco (Miami, FL, USA)/ThermoFisher (Waltham, MA, USA), catalog number 15140122; and 10% FBS, Fetal Bovine Serum, from Biowest, Nuaillé, France, catalog number S1810) at a temperature of 37 °C for 48 h. 

Cells were seeded at a density of 30,000 cells/cm^2^ in 96-well plates and were incubated at 37 °C in a 5% CO_2_ Sanyo MCO19AICUV incubator for 24 h. The extracts were used at the initial concentration of 20 mg/mL and were also diluted to obtain equivalent extract concentrations of 10, 5, 2.5, and 1.125 mg/mL. Each of the concentrations was tested four times. Two controls were set up: a negative control, where cells were cultured with a complete culture medium, and a positive control, where the culture medium was supplemented with 10% dimethyl sulfoxide, a cytotoxic compound. After 48 h of incubation, the cell culture media were aspirated and replaced by a medium containing 50% of the complete culture medium and 50% of a 0.04 mg/mL resazurin solution prepared using a phosphate-buffered saline (PBS) solution. After 3 h of incubation at 37 °C and 5% CO_2_, the absorbance of each well was measured at 570 and 600 nm, which correspond to the absorbance maxima of resorufin and resazurin, respectively. Metabolically active cells reduce resazurin to resorufin and the conversion rate is assumed to be proportional to the cell population. The OriginPro 2018 software was used to perform analysis of variance (ANOVA) to determine the significance of differences between samples for each concentration. Tukey’s test was used for multiple comparisons, and the differences were assumed to be statistically significant if *p* < 0.05. Cell viability is given as a percentage of viable cells in the samples to test relative to the negative control:

(2) $$\%cell\;viability=\frac{treated\;cells}{control\;cells}\times 100$$

## 3. Results and Discussion

### 3.1. Critical Micelle Concentration (CMC)

**Figure 2:**
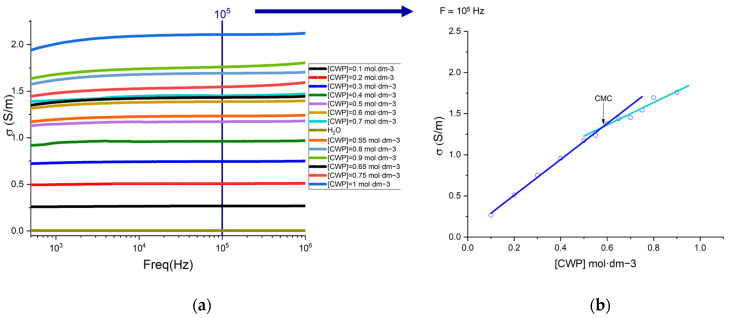
(**a**) Conductivity vs. frequency for different concentrations of the CWP solutions. The different concentrations are represented in the graph in the form of [CWP] = “concentration of the solution” mol·dm^−3^. (**b**) CMC vs. CWP concentration; f = $10^{5}$ Hz.

The critical micelle concentration (CMC) is defined as a phenomenon that separates two distinct behaviors of the size distribution of micelles [31]. The surfactants are characterized as amphiphilic molecules that comprise two different parts, polar and hydrophilic [31]. In water, these molecules present a specific organized molecule configuration, defined as micelles. Nevertheless, this phenomenon is only verified for concentrations above the CMC. It is represented by an inflection point that can be calculated through the increase in the concentration of amphiphilic molecules, which results in changes in the physicochemical properties of the surfactant solution [32]. Therefore, with the selected technique, the synthesis of the nanocomposite can only be verified if the concentration of the CWP in the solution has a CMC value. As seen in Figure 2, it is possible to analyze the variation of conductivity as a function of frequency for the different concentrations of CWP under study. Figure 2a shows the variation of the conductivity for the different concentrations of CWP, which goes from 0.1 mol/dm^3^ to 0.9 mol/dm^3^. For a comparative study, $10^{5}$ Hz was chosen as the reference frequency. Figure 2b represents the variation of conductivity as a function of the concentration of the CWP solutions, for a frequency of $10^{5}$ Hz. The inflection point, in this case, is the intersection point of the two lines resulting from the linear fittings performed, representing the CMC value. Thus, a value of 0.58 mol/dm^3^ was obtained. 

### 3.2. Thermal Analysis

**Figure 3:**
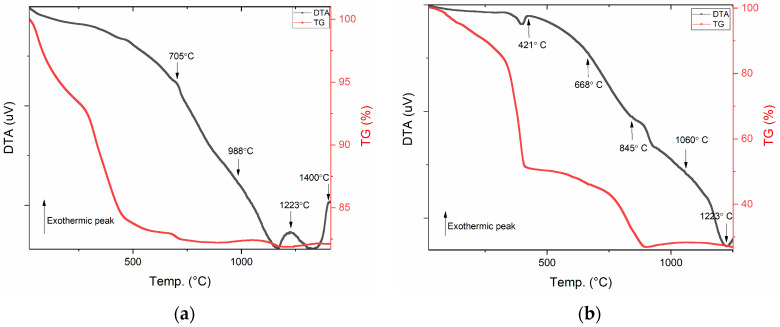
DTA and TG of (**a**) Gd_3_Fe_5_O_12_ and (**b**) ZnFe_2_O_4_ samples.

Figure 3 shows the results of the differential thermal analysis (DTA) and thermogravimetric analysis (TG) performed on the Gd_3_Fe_5_O_12_ and ZnFe_2_O_4_ powders after the first heat treatment at 350 °C. In these thermograms, the exothermic peaks without associated weight loss are commonly related to phase crystallization.

In Figure 3a, thermal analysis of Gd_3_Fe_5_O_12_ sample, it is possible to highlight three exothermic peaks at 705, 988, and 1223 °C without associated weight loss. Thus, suggesting that for the indicated temperatures, it is possible for meaningful structural variation to occur. Regarding the ZnFe_2_O_4_ sample, (Figure 3b), five exothermal peaks without any loss of mass can be verified, making them relevant to analyze. The peaks that suggest the existence of structural changes are centered at 421, 668, 845, 1060, and 1220 °C. Thus, the heat treatments that will be applied to the Gd_3_Fe_5_O_12_ and ZnFe_2_O_4_ samples will be for temperatures of 500, 700, 850, 1000, 1200, and 1400 °C. 

### 3.3. Morphological and Structural Characterization

**Figure 4:**
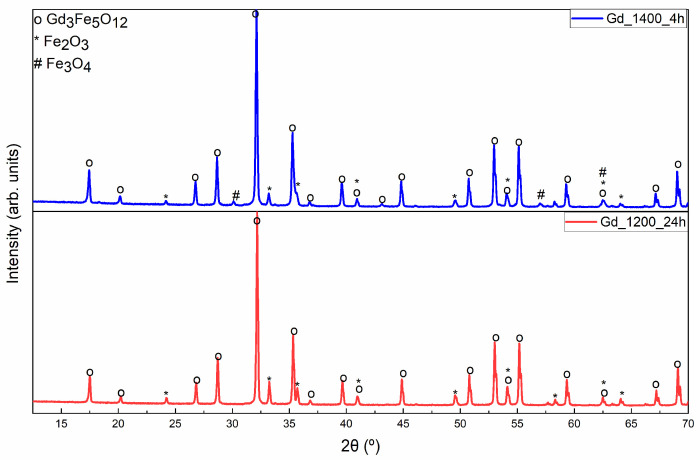
XRD diffractograms of the Gd_3_Fe_5_O_12_ samples heat-treated at 1200 °C for 24 h (red line) and at 1400 °C for 4 h (blue line).

Figure 4 illustrates the diffractograms taken for the different Gd_3_Fe_5_O_12_ samples. In Figure 4, we only presented the phases of HT at 1200 and 1400 °C since they were the only ones with a composition of Gd_3_Fe_5_O_12_. Other than that, different times of HT (4 h and 24 h) were chosen to analyze the impact of the heating rate on the samples’ composition. It is possible to conclude that none of the samples had a pure Gd_3_Fe_5_O_12_ composition. Fe_2_O_3_, Fe_3_O_4_, and Gd_3_Fe_5_O_12_ phases were identified. The sample with the highest Gd_3_Fe_5_O_12_ composition was heat-treated at 1200 °C for 24 h. The sample heat-treated at 1200 °C for 4 h is not presented because it has secondary crystal phases (GdFeO_3_, Fe_2_O_3_, and Fe_3_O_4_) and a high crystallite size.

**Figure 5:**
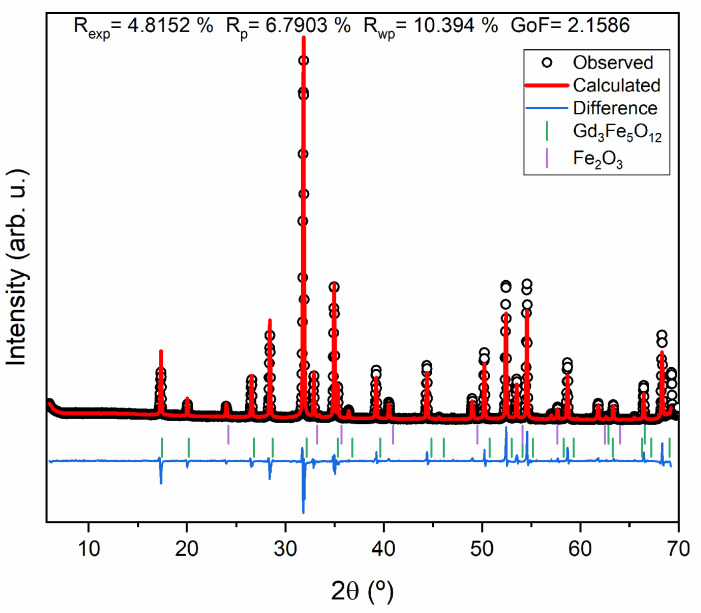
Rietveld refinement of the Gd_3_Fe_5_O_12_ sample heat-treated at 1200 °C for 24 h.

A Rietveld refinement was applied to the sample with the highest Gd_3_Fe_5_O_12_ composition (HT at 1200 °C for 24 h). The Goodness of Fit (GoF) and the ratio of R_wp_ to R_exp_ are two parameters that, according to the literature, are used to examine the quality of Rietveld refinement [33,34]. By looking at the parameters presented in Figure 5, it is possible to confirm that R_wp_ > R_exp_ and that the GoF is ≈2, which demonstrates a good refinement quality.

**Figure 6:**
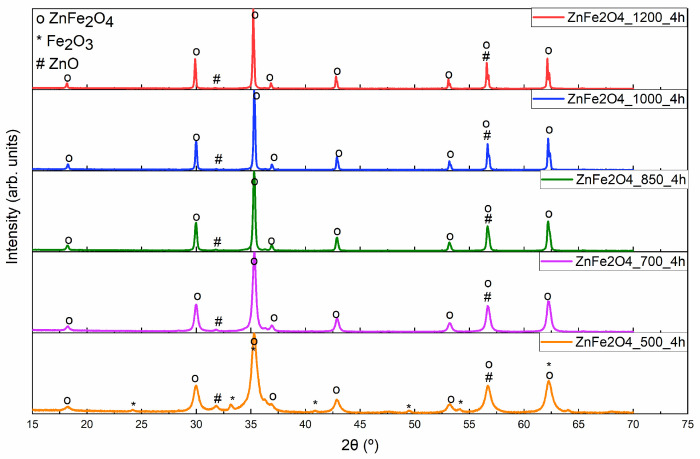
XRD of the ZnFe_2_O_4_ samples heat-treated at 500, 700, 850, 1000, and 1200 °C for 4 h.

**Figure 7:**
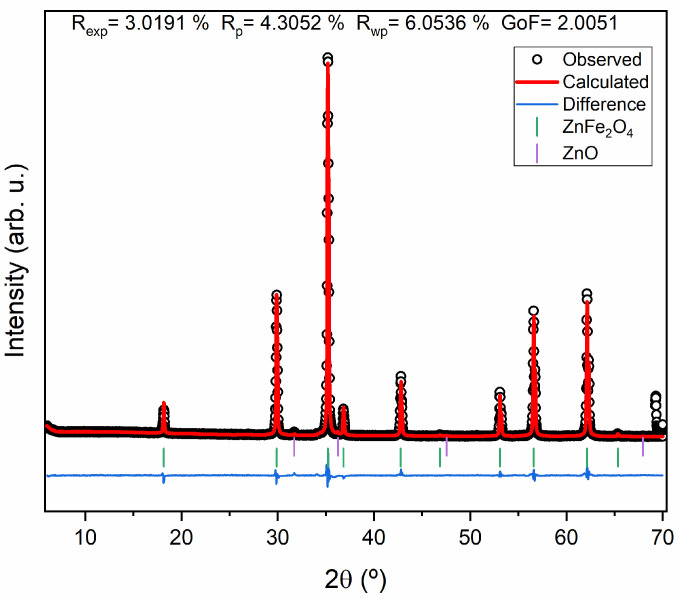
Rietveld refinement of the sample of ZnFe_2_O_4_ heat-treated at 1200 °C for 4 h.

Figure 6 illustrates the XRD of the ZnFe_2_O_4_. It should be noted that none of the synthesized samples show a pure composition of ZnFe_2_O_4_ since different phases of ZnO and even Fe_2_O_3_ have been identified. However, the sample that exhibits a higher ZnFe_2_O_4_ composition (97%) is the sample heat-treated at 1200 °C for 4 h. Figure 7 shows the Rietveld refinement fit, where the R_wp_ parameter value is higher than the R_exp_ and has a GoF of 2.0051.

**Figure 8:**
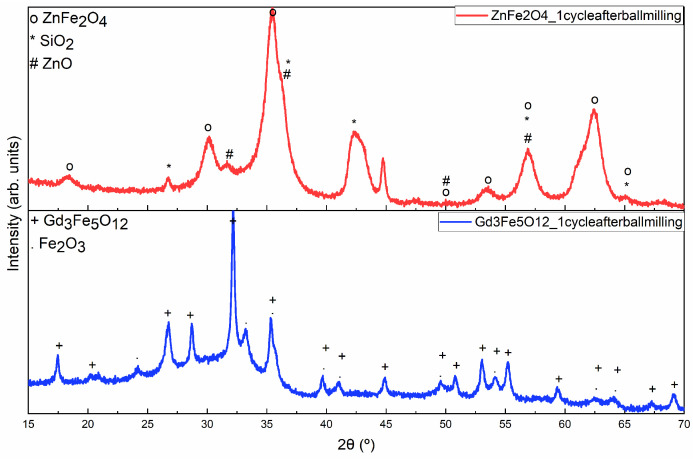
XRD diffractograms of the ZnFe_2_O_4_ (red line) and Gd_3_Fe_5_O_12_ (blue line) samples after one cycle of ball milling.

In comparative terms, after performing a ball-milling procedure to decrease the particle size, an X-ray diffractogram was conducted on both samples with the highest composition of Gd_3_Fe_5_O_12_ (HT 1200 °C for 24 h) and ZnFe_2_O_4_ (1200 °C for 4 h). Observing Figure 8, it can be noticed that in the ZnFe_2_O_4_ sample, a SiO_2_ contamination is verified, probably caused by the agate grinding balls used for ball milling [35]. One explanation for this contamination not occurring in the Gd_3_Fe_5_O_12_ sample may be due to the intensity of the peaks, which are much more intense in the case of the Gd_3_Fe_5_O_12_ sample. Also, both diffractograms reveal some amorphous phase due to the presence of SiO_2_.

**Figure 9:**
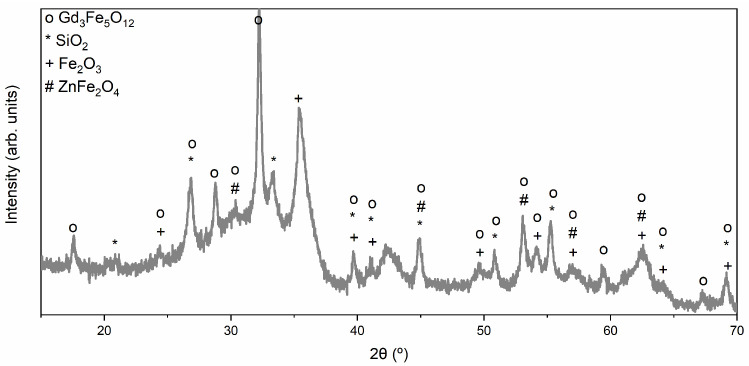
XRD diffractograms of the produced composite.

The phases with the highest composition of Gd_3_Fe_5_O_12_ (HT 1200 °C for 24 h) and ZnFe_2_O_4_ (1200 °C for 4 h) were chosen to synthesize the composite. The structural analysis performed on the composite, illustrated in Figure 9, shows that four different phases are present, in agreement with the results obtained previously: Gd_3_Fe_5_O_12_, ZnFe_2_O_4_, Fe_2_O_3_, and SiO_2_ (from the agate ball contamination). The composite presents a composition of 65% Gd_3_Fe_5_O_12_ and 10% ZnFe_2_O_4_. The Rietveld refinement obtained for the nanocomposite showed a GoF of 1.78 and a higher R_wp_ than R_exp_.

**Figure 10:**
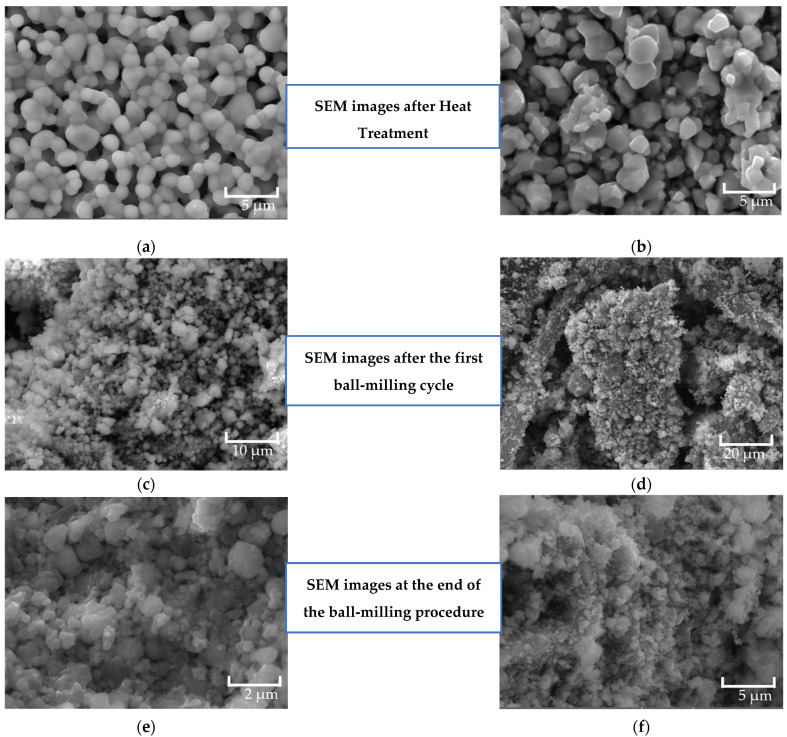
SEM analysis performed on the (**a**) Gd_3_Fe_5_O_12_ (HT 1200 °C 24 h) and (**b**) ZnFe_2_O_4_ (HT 1200 °C 4 h) samples after heat treatment. These samples were used for the synthesis of the nanocomposite. (**c**) Gd_3_Fe_5_O_12,_ and (**d**) ZnFe_2_O_4_, represent the samples after the first ball-milling cycle performed. These images were used to evaluate if it was necessary to execute another cycle of ball milling. In the end, after the total ball-milling procedure, (**e**) Gd_3_Fe_5_O_12_ and (**f**) ZnFe_2_O_4_ were obtained.

Regarding the morphological analysis performed on the samples, Figure 10 illustrates the SEM analysis performed on the Gd_3_Fe_5_O_12_ (Figure 10a) and ZnFe_2_O_4_ (Figure 10b) samples after the heat treatment; Figure 10c,d represents the samples after the first ball-milling cycle performed on the Gd_3_Fe_5_O_12_ (Figure 10c) and ZnFe_2_O_4_ (Figure 10d) samples; and Figure 10e,f shows the results obtained at the end of the ball-milling process of the Gd_3_Fe_5_O_12_ (Figure 10e) and ZnFe_2_O_4_ (Figure 10f) samples.

Figure 10 shows the Gd_3_Fe_5_O_12_ particles after heat treatment, with large aggregates of circular structures, as described by Jiang L. et al. [34]. The average particle size determined by ImageJ 1.52v software is 1.09 μm. The SEM images obtained for the ZnFe_2_O_4_ sample are significantly irregular, with large agglomerates. The average size of the synthesized particles is approximately 1.59 μm. Firstly, these micrographs were obtained with pellets of the samples. Only after the morphological analysis were the pellets crushed.

In comparative terms, after conducting the first ball-milling cycle, each sample was analyzed to verify the size and uniformity. In both Figure 10c,d, it is possible to conclude that both samples are significantly irregular, especially in terms of grain size uniformity. The average grain size is 0.92 μm for the Gd_3_Fe_5_O_12_ sample (Figure 10c) and 0.51 μm for the ZnFe_2_O_4_ sample (Figure 10d). Due to their irregularity in terms of grain size distribution, it was decided that it was necessary to execute one more cycle of ball milling.

Therefore, micrographs in Figure 10e,f were acquired after the total ball-milling process for Gd_3_Fe_5_O_12_ and ZnFe_2_O_4_, respectively. The results allow us to emphasize the presence of agglomerates in both samples. The Gd_3_Fe_5_O_12_ particles, as seen in Figure 10e, present an average particle size of 273 nm, and the ZnFe_2_O_4_ samples have an average size of 196 nm. These two samples were used for the synthesis of the nanocomposite, with the help of a planetary ball milling, to make their mixing possible.

**Figure 11:**
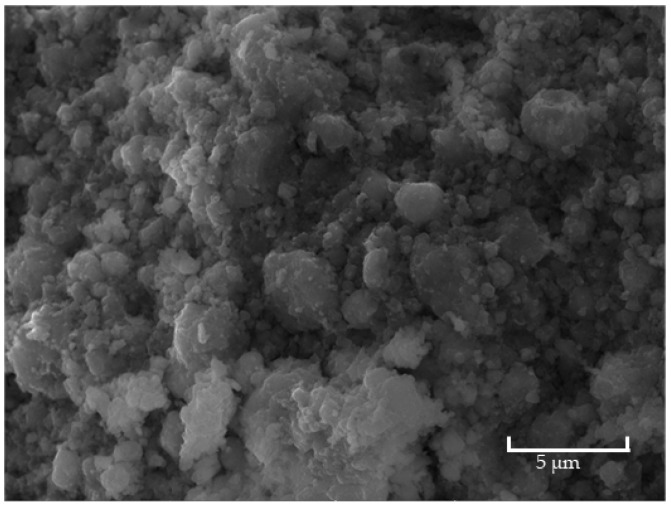
SEM analysis performed on the synthesized composite.

The morphology of the synthesized composite is shown in Figure 11, where the presence of large agglomerates is clearly visible. The average particle size obtained for the composite was 155 nm.

### 3.4. Magnetic Characterization

**Figure 12:**
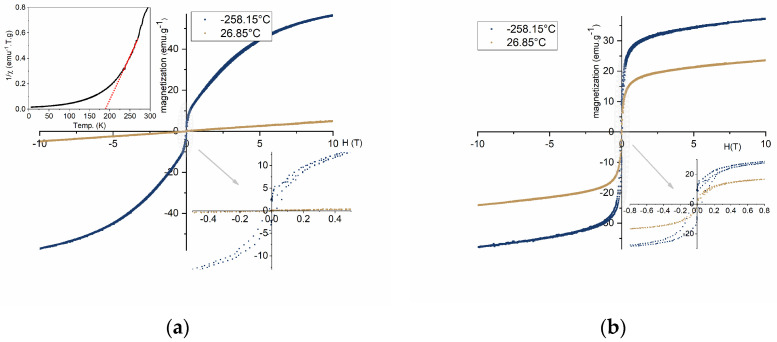
(**a**) Hysteresis loop and the dependence of the inverse with temperature (top inset) for Gd_3_Fe_5_O_12_ and (**b**) hysteresis loop of ZnFe_2_O_4_ samples tested at different temperatures.

Figure 12a represents the hysteresis M–H curve for Gd_3_Fe_5_O_12_ sample at temperatures of −258.15 and 26.85 °C, i.e., room temperature. The sample tested at −258.15 °C has a saturation magnetization of 57.03 emu/g, denoting a dominant ferromagnetic behavior, as represented in Figure 12a. This behavior is in accordance with what is reported in the literature [36]. The sample tested at 26.85 °C, on the other hand, does not enable us to calculate the value of saturation magnetization since its magnetization rises with increasing values of the magnetic field, demonstrating no magnetic hysteresis. The magnetization’s tendency to increase linearly with the magnetic field can be attributed to a typical behavior of paramagnetic nanoparticles (Figure 12a) [34]. Additionally, analyzing the inverse of susceptibility with the variation of temperature (top inset of Figure 12a), above −272.15 °C, the sample demonstrates a variation from a ferromagnetic to paramagnetic behavior, corroborating the previous analysis. This temperature-dependent change in the magnetic behavior is a direct consequence of the paramagnetic contribution associated with Gd^3+^ and the antiferromagnetic contribution from the octahedral structure of the Fe sublattice [37]. Due to the magnetic unit cell’s ordered spins in the spin-canted FeO_6_ octahedra structure, the antiferromagnetic contribution was prominent at low temperatures. The ferromagnetism gradually diminished as the surrounding temperature increased and the paramagnetic contribution of Gd^3+^ ions took control [34].

The hysteresis curve for the ZnFe_2_O_4_ sample is shown in Figure 12b. First, it is clear that the nanoparticles have a larger saturation magnetization at −258.15 °C, with a value of roughly 37.16 emu/g, compared to the 23.67 emu/g obtained for a temperature of 26.85 °C. However, both samples exhibit ferromagnetic behavior in both circumstances. According to the literature [37], it is described that particles with a diameter between 29 and 35 nm, at room temperature present ferromagnetic behavior with clear evidence of a hysteresis curve. In the first instance, in terms of the comparison of the magnetic behavior of this present work, despite the difference in the particle size, its behavior is similar to what is described in the literature [37]. Regarding the values of saturation magnetization obtained for the synthesized particles, compared to that described in the literature [37], approximately 10 emu/g, it assumes a significantly higher value, which is noted to be quite favorable.

**Figure 13:**
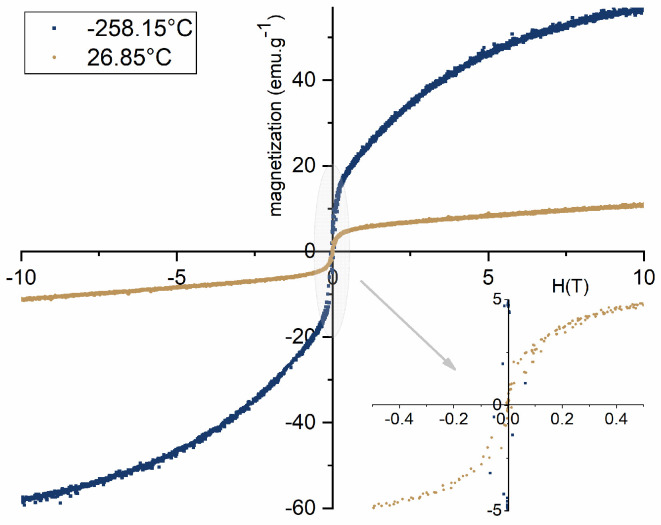
Hysteresis loop for composite samples tested at different temperatures.

The examination of the M–H curve of the composite, as seen in Figure 13, obtained by the mixture of Gd_3_Fe_5_O_12_ and ZnFe_2_O_4_, allows us to infer that, as previously proven, the saturation magnetization has greater values at −258.15 °C, according to what was expected. Secondly, in terms of magnetic behavior, it is noted that due to the presence of ZnFe_2_O_4_ in the composite, the particles behave ferromagnetically at both analysis temperatures. While the saturation magnetization for −258.15 °C was 57.30 emu/g, the result for 26.85 °C was 11.56 emu/g. The low saturation magnetization and ferromagnetic behavior of the synthesized composite at room temperature can be explained by the constitution of the sample (XRD analysis (Figure 9)). At room temperature, the paramagnetic behavior from Gd_3_Fe_5_O_12_ with the dominant ferromagnetism behavior from ZnFe_2_O_4_ and the antiferromagnetism from Fe_2_O_3_ [38] result in a magnetization with values lower than the obtained for ZnFe_2_O_4_ but slightly higher than the ones obtained for Gd_3_Fe_5_O_12_.

Individually, the SAR obtained for the synthesized Gd_3_Fe_5_O_12_ sample was $0.3\pm 0.2\;(W\cdot g^{-1})$. By itself, the SAR value obtained is lower than the ambitioned. The size of the nanoparticles, their state of aggregation, and their composition are some of the variables that might be at the root of this considerable variation. The SAR value obtained for the produced ZnFe_2_O_4_ nanoparticles was approximately 0 (W/g). The low SAR value obtained, compared to the literature [39], may be due to the agglomeration of the particles, resulting in the increase in the average particle size (Figure 11) compared with the ones referred to in the literature [39]. Due to their limited thermal efficiency, these particles do not appear to be feasible for use in magnetic hyperthermia on their own according to the data obtained. However, their low SAR value may be due to their high particle size, and they may have greater values if a size decrease could be achieved.

Finally, the SAR analysis of the synthesized nanocomposite obtained a value of $0.5\pm 0.2\;(W\cdot g^{-1})$. Firstly, the obtained SAR value is higher than the values obtained for Gd_3_Fe_5_O_12_ and ZnFe_2_O_4_. The composite’s SAR value would be predicted to be lower than the one obtained for the Gd_3_Fe_5_O_12_ sample due to its composition of 65% Gd_3_Fe_5_O_12_ and 10% ZnFe_2_O_4_. However, the obtained SAR value allows us to conclude that the mixing of these two crystal phases resulted in a composite with superior thermal efficiency. In this approach, and despite the low value attained, the nanocomposite appears to be more feasible for magnetic hyperthermia treatment than Gd_3_Fe_5_O_12_ and ZnFe_2_O_4_ particles alone.

### 3.5. Biological Analysis

**Figure 14:**
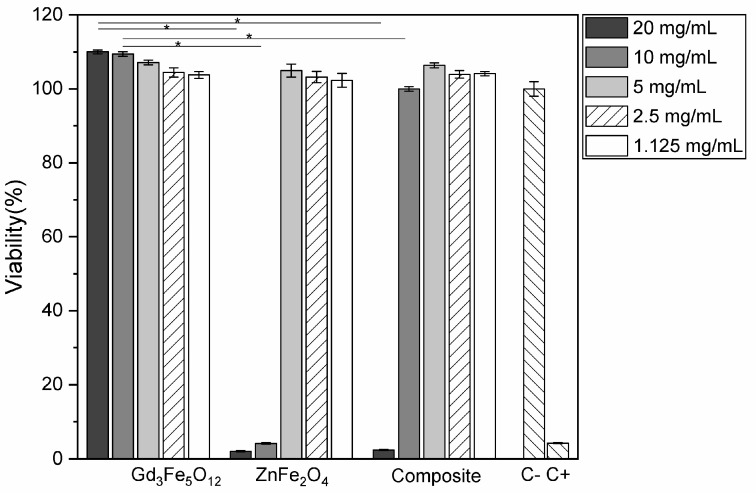
The cell viability of synthesized powders as a function of concentration. The composite is a mixture of Gd_3_Fe_5_O_12_ and ZnFe_2_O_4_. C− and C+ is the negative and positive control, respectively. The results were statistically compared for each concentration between each sample, using ANOVA with a significance level of *p* < 0.05 (represented by an asterisk).

To evaluate the cytotoxicity of the samples, the extract method was used based on different extract concentrations. Analyzing the three different compounds, ZnFe_2_O_4_ is the one that presents the highest cytotoxicity. In the case of the synthesized Gd_3_Fe_5_O_12_ particles, it is worth noting that they appear to have a non-cytotoxic behavior for all the studied concentrations. ZnFe_2_O_4_ particles appear to be non-cytotoxic only for concentrations lower than 5 mg/mL. The composite exhibits cytotoxic behavior at concentrations of 20 mg/mL. This behavior is a consequence of the strong cytotoxic effect of ZnFe_2_O_4_ (Figure 14).

The particles, when in contact with culture media, release ions and present a low dissolution rate due to their high crystallinity. This type of particle can be used not only for MH but also for bone regeneration since the release of gadolinium ions can improve biocompatibility, osteoconductivity, and osteoinductivity [40].

## 4. Conclusions

This study focuses on a novel composite made of gadolinium iron garnet (Gd_3_Fe_5_O_12_) and zinc ferrite (ZnFe_2_O_4_). The nanocomposite was created using an eco-friendly coconut water-assisted sol–gel method. The purest phase of each ferrite (Gd_3_Fe_5_O_12_ and ZnFe_2_O_4_) was used to guarantee the purest composition and the best magnetic properties of the nanocomposite. With an average particle size of 155 nm, the composite exhibits ferromagnetic behavior with a saturation magnetization of 11.56 emu/g and a specific absorption rate (SAR) of 0.5 ± 0.2 (W/g). Cytotoxicity tests showed no harmful effects at doses below 10 mg/mL. While its efficiency for magnetic hyperthermia application is poor, the saturation magnetization values obtained for the nanocomposite denote its importance in being used for biomagnetic applications.

## Data Availability

Data are contained within the article.
